# Blood pressure response to exercise in children and adolescents

**DOI:** 10.3389/fcvm.2022.1004508

**Published:** 2022-09-30

**Authors:** Julio Alvarez-Pitti, Vesna Herceg-Čavrak, Małgorzata Wójcik, Dragan Radovanović, Michał Brzeziński, Carl Grabitz, Elke Wühl, Dorota Drożdż, Anette Melk

**Affiliations:** ^1^Pediatric Department, Consorcio Hospital General, University of Valencia, Valencia, Spain; ^2^CIBER Fisiopatología Obesidad y Nutrición (CIBEROBN), Instituto de Salud Carlos III, Madrid, Spain; ^3^INCLIVA Biomedical Research Institute, Hospital Clínico, University of Valencia, Valencia, Spain; ^4^Faculty of Health Science, Libertas International University, Zagreb, Croatia; ^5^Department of Pediatric and Adolescent Endocrinology, Chair of Pediatrics, Pediatric Institute, Jagiellonian University Medical College, Kraków, Poland; ^6^Department of Medical Sciences, Faculty of Sport and Physical Education, University of Niš, Niš, Serbia; ^7^Department of Pediatrics, Gastroenterology, Allergology and Pediatric Nutrition, Medical University of Gdansk, Gdańsk, Poland; ^8^Children’s Hospital, Hannover Medical School, Hanover, Germany; ^9^Division of Pediatric Nephrology, Center for Pediatrics and Adolescent Medicine, Heidelberg University Hospital, Heidelberg, Germany; ^10^Department of Pediatric Nephrology and Hypertension, Pediatric Institute, Jagiellonian University Medical College, Kraków, Poland

**Keywords:** blood pressure (BP), exercise, stress test, children, adolescents, arterial hypertension, cardiovascular risk

## Abstract

Blood pressure changes during exercise are part of the physiological response to physical activity. Exercise stress testing can detect an exaggerated blood pressure response in children and adolescent. It is applied for certain clinical conditions, but is also commonly used as part of the assessment of athletes. The interpretation of blood pressure values in response to exercise during childhood and adolescence requires appropriate reference data. We discuss the available reference values and their limitations with regard to device, exercise protocol and normalization. While the link between an exaggerated blood pressure response and cardiovascular events and mortality has been demonstrated for adults, the situation is less clear for children and adolescents. We discuss the existing evidence and propose that under certain circumstances it might be reasonable to have children and adolescents undergo exercise stress testing as a rather non-invasive procedure to add additional information with regard to their cardiovascular risk profile. Based on the existing data future studies are needed to extend our current knowledge on possible links between the presence of certain clinical conditions, the detectability of an exaggerated blood pressure response during childhood and adolescence and the risk of developing cardiovascular morbidity and mortality in later life.

## Introduction

The changes in blood pressure (BP) during exercise are mainly seen as part of the physiological toward an increased demand. Standardized exercise protocols are used as part of certain clinical investigations, but also to assess cardiorespiratory fitness in athletes. The interpretation of BP values in children and adolescents upon exposure to different protocols is hampered by the scarcity of respective reference values. While data from adults suggest a relationship between an exaggerated BP response to exercise and the risk of developing arterial hypertension (HTN), the situation is less clear in children and adolescents.

This narrative review intends to provide an overview of the physiology behind the BP changes detected during exercise, the available reference data for children and adolescents to assess BP after engagement in different exercise protocols, potential clinical conditions that may predispose toward an exaggerated BP response as well as the implications such an exaggerated BP response might have.

## Physiology of blood pressure changes during exercise

**FIGURE 1:**
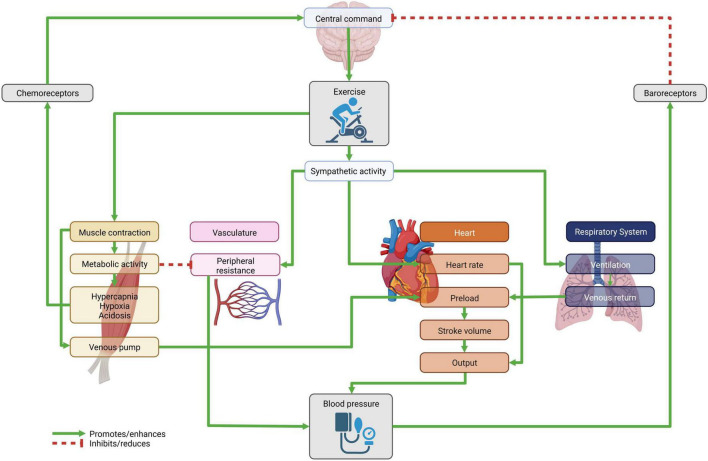
Feedback loop as the physiological response to physical activity. The central command initiates muscle activity via somatomotor neurons. Simultaneously the vascular, heart and respiratory systems are activated, in part through the increasing sympathetic activity. This leads to a surge in cardiac output and peripheral vascular resistance, which in turn raises (systolic) blood pressure. The change in hemodynamics triggers the baroreceptor reflex, while the metabolic activity of muscle exertion is sensed by chemoreceptors. Figure created with BioRender.com.

Blood pressure changes during exercise are part of the complex physiological response of the cardiovascular (CV) system to physical activity (Figure 1). To preserve cellular oxygenation and acid-base homeostasis during exercise, metabolic, CV, and respiratory responses must adapt rapidly to these changes. This task is accomplished by increases of heart rate and stroke volume and decreases in systemic as well as pulmonary vascular resistance. Precise control mechanisms are required for this process to balance systemic vascular resistance (SVR) in context of metabolic vasodilation at the muscle. In general, the increment in cardiac output with exertion is larger than the rise in mean systemic BP reflecting a decrease in SVR (1). The ability to decrease SVR has been correlated with exercise performance (2). Changes in the sympathetic and parasympathetic nervous systems are responsible for the CV adjustment during exercise such as increased cardiac output, skeletal muscle flow, and BP. Damage to the neurophysiological mechanisms could result in an inadequate blood supply to the muscle and the brain. Circulation control and control of the BP is a multifactorial process and complex interaction of peripheral reflexes (exercise pressor reflex, arterial and cardiopulmonary baroreflexes) and higher brain centers (central command, see below). During mild to moderate exercise in healthy subjects, BP is well maintained by increasing heart rate and cardiac output. With increased duration and intensity exercise is accompanied by vasoconstriction in many organs, especially the splanchnic region and kidneys (3). The exercise pressor reflex (EPR), which is the most important peripheral reflex response during physical activity, consists of two components: the mechanoreflex and the metaboreflex. Afferent fibers from skeletal muscle of groups III (mechanoreceptors) and IV (metaboreceptors) are excitable by mechanical and chemical stimulation (4). Mechanoreceptors are activated by mechanical deformation induced by pressure or stretch. Metaboreceptors are sensitive neural fibers activated by metabolites (lactic acid, potassium, bradykinin, arachidonic acid products etc.) accumulating in the muscle during contraction. Although mechano- and metaboreceptor stimulation occur mainly simultaneously, activation is different in different types of physical activity, i.e., reflecting dynamic or static exertion. Mechano- and metaboreflex also interact with other reflexes induced by baroreceptors and chemoreceptors. Baroreceptors, located in the carotid sinuses and aortic arch, are a part of the vascular system’s autoregulation toward hemodynamic changes. They reduce fluctuations in arterial pressure by lowering sympathetic activity as arterial pressure rises. During the exercise, however, the baroreceptor reflex is not inhibited or overridden, but is reset and allows increases in BP, heart rate and sympathetic activity (5). In addition to reflex stimuli, the central neural mechanism so-called *central command* is involved in controlling the CV response to the muscular effort. Neurons of the motor cortex together with the medulla oblongata activate the CV response to exertion (6). Their role is to coordinate and transmit excitatory impulses to descending somato and locomotor neurons in parallel to activate the somatomotor-, respiratory- and CV systems simultaneously. Alterations in these control mechanisms could result in an inadequate blood supply to the muscle and the brain.

Changes in the sympathetic and parasympathetic nervous systems are responsible for the CV adjustment during exercise such as increased cardiac output, skeletal muscle flow, and BP. The hemodynamic status during dynamic exercise is regulated by integrated signals from the brain and periphery, which increases the activity of the sympathetic and reduces the parasympathetic nervous system. As a consequence, heart rate, myocardial contractility, stroke volume, and cardiac output are increased. At the same time, significant metabolic vasodilation and a decrease in SVR occur in the large muscles involved in physical activity.

Another important factor contributing to the regulation of BP during exercise is process of vasodilation. The key regulator of that process is Nitric oxide (NO). It is a soluble gas continuously synthesized from the amino acid L-arginine in endothelial cells by the constitutive calcium-calmodulin-dependent enzyme nitric oxide synthase (eNOS). This substance has a wide range of biological properties that maintain vascular homeostasis, including modulation of vascular dilator tone, regulation of local cell growth, and protection of the vessel from injurious consequences of platelets and cells circulating in blood, playing in this way a crucial role in the normal endothelial function (7). It has been shown, that as a response to the mechanical stimuli the activity of eNOS is increasing (8). A recently published study found that response to nitric oxide production may vary with age. The vasodilatory response to 30 min of cycling at 60% $\dot{\text{V}}$ O2_max_ was greater and occurred earlier in boys compared with men (9). The beneficial effect of different types of physical activity on endothelial function in both adults and children and adolescents is known (10, 11). But preliminary studies indicate that even in children and adolescents with some degree of endothelial dysfunction (such as children with obesity or with abnormal BP), physical activity improves endothelial function (12–14).

Mean arterial pressure (MAP) is therefore only slightly elevated during physical activity, diastolic pressure does not change, while systolic pressure increases during exercise. Vasoconstriction occurs in organs and tissues that are not directly involved in the exercise. This arterial vasoconstriction in non-exercising organs together with the activation of the muscle and respiratory pump and sympathetic venoconstriction increases cardiac preload and consequently stroke volume (6).

The CV response toward static exercise differs significantly from that observed during dynamic exercise. During static exercise, in contrast to dynamic, there is no significant vasodilation in the muscles due to intramuscular pressure produced by isometric contractions. Peripheral systemic resistance decreases but not as much as in dynamic exercise. Cardiac output increases during static contractions due to increased heart rate. The increased cardiac output without a significant decrease in peripheral vascular resistance causes an increase in BP with increases in systolic BP, diastolic BP and MAP. These changes occur in an attempt to maintain adequate perfusion of muscle during static exercise (15). Stroke volume is relatively constant during low-intensity contractions and decreases during high-intensity contractions. It is probably the result of decreased preload because of high intrathoracic pressure, and an increased afterload. Static exercise is characterized by a rapid increase in both systolic and diastolic pressure, termed the *pressor response*, which appears to be inappropriate for the amount of work produced by the contracting muscle. Compared to the slight increase in MAP during dynamic exercise, MAP is markedly elevated in static exercise (16). Considering the described pathophysiological mechanism of static loading in exercise, traditionally aerobic exercise has been shown to be beneficial in lowering blood pressure. It was recommended that children do not engage in sports with predominantly static loading until they have completed their physical and skeletal maturity. This particularly applied to children with uncontrolled hypertension, seizure disorders, pulmonary hypertension, and children treated with cardiotoxic drugs (17). However, a positive effect of static load on arterial pressure values was observed in adults. Although the positive effects of resistance training on BP values was registered in adults there is limited understanding about the static loading on BP in children and adolescents. The recently published meta-analysis of Guillem et al. (18) did not clearly confirm the positive effect of static exercise in young people on BP, but a non-significant decrease in both, systolic and dyastolic BP was observed. Authors explained that although there is limited research in this topic, the results of their research suggest that “resistance training does not have an adverse effect on the BP of children and adolescents and may be beneficial in lowering BP and improving BMI in this population.” In general, today’s scientific research supports the acceptance that children and adolescents can participate in static sports if the activities are performed with an emphasis on proper technique and under good supervision (19). Nevertheless, and following the recommendations of the American Academy of Pediatrics, “athletes with poorly controlled, preexisting hypertension require consultation with a medical professional because of the risk of marked elevation of BP during resistance training with weights. Using one’s own body weight is an acceptable alternative until a consultation can be obtained” (20).

After the exercise is terminated, the impulses of the metabo- and mechanoreceptors cease as well as the stimulus from the central command. This leads to a decrease in sympathetic activity and an increase in parasympathetic activity. Consequently, heart rate, myocardial contractility, stroke volume, cardiac output, and MAP rapidly decline. Cessation of muscle pump activity leads to decreased cardiac filling resulting in a decrease in stroke volume and the consequent reduction in MAP if baroreflex-mediated peripheral vasoconstriction does not contemporary adjust heart rate and SVR (4).

A phenomenon called post-exercise hypotension refers to a sustained decrease in BP after a single episode of exercise (21). Post-exercise hypotension is consistently elicited after longer (30–60 min) bouts of moderate-intensity exercise (22). The hypotensive effect during the post- period is observed in both, hypertensive and normotensive subjects, and may last nearly 120 min (23). The mechanisms responsible for BP reduction after aerobic training are not clear. Hypertension has a multifactorial etiology and, therefore, several mechanisms may be involved in the hypotensive effects of aerobic training. The cause of the vasodilatation underlying post-exercise hypotension in humans remains largely unexplained (24). It does not depend on the decease of adrenergic receptor responsiveness, neither on nitric oxide level (23). It has been suggested, that the phenomenon may be associated with ineffective transduction of sympathetic nerve activity into vasoconstriction and lesser neurotransmitter release (25). Sympathetic nerve terminals possess pre-synaptic inhibitory opioid receptors that may be occupied after exercise, effectively reducing noradrenaline release (26). Although systemic opioid blockade with naloxone has reversed post-exercise hypotension in animals, the role of opioids in humans remains controversial (27, 28). Pre-synaptic inhibition can also be caused by noradrenaline via a2-adrenergic receptor activation or by neuropeptide Y, which is co-released with noradrenaline during exercise (29). After exercise, neuropeptide Y may remain bound to pre-synaptic receptors, reducing noradrenaline release. None of these observations provide a complete and unequivocal explanation of the phenomenon.

The autonomic nervous system may provide useful information about the functional adaptations of the body. The heart rate adaption to exercise training is the result of changes in autonomic tone. The heart rate of trained persons at rest is lower than that of untrained due to the dominance of the parasympathetic tone. During exercise, trained individuals can achieve appropriate SBP values with a lower heart rate due to a higher stroke volume. Diastolic pressure values do not differ significantly. Trained athletes have a higher relative maximum oxygen consumption during physical effort, a lower resting heart rate and a faster short-term and long-term heart rate recovery than untrained people. Rapid recovery after exercise involves a coordinated interaction of parasympathetic reactivation and sympathetic withdrawal. Delayed heart rate recovery has been shown to be a strong predictor of mortality (30).

## Evaluation of blood pressure response to exercise

During submaximal and maximal intensity exercise, a moderate increase in BP is expected among children and adolescents. However, there are very little specific data on BP changes depending on the duration and intensity of physical activity. Standardized load testing involving cycle ergometers or treadmills is used to evaluate the CV system. These exercise tests are done in specially equipped laboratories and are meaningful for certain clinical conditions, but are also used as part of the assessment of athletes. Table 1 gives an overview of the publications that provide reference data for BP response toward different exercise protocols.

**TABLE 1:** Overview of studies reporting reference values for blood pressure during exercise testing in childhood.

Author	Age	N (m/f)	Country	Selection	Device	Protocol	Termination	Normalization	Adjustment	Limitations
Clarke et al., 2021 (31)	6–18 y	648 (314/334)	Australia	Single center routine examination, normal cardiac anatomy, BMI < P95, BP < P95	Treadmill	Bruce protocol	Exhaustion	P5, P10, P50, P90, P95 of SBP change	Age, sex, height	Reports only change of SBP from baseline
Sasaki et al., 2021 (34)	7–17 y	1085 (642/397)	USA	Single center routine examination, normal cardiac anatomy	Treadmill	Modified Bruce protocol	Exhaustion	Table for P5, P10, P50, P90, P95 of SBP/DBP Formula by linear regression for P50, P90, P95 of SBP	Age, sex	No adjustment for height
Burstein et al., 2021 (59)	6–18 y	1829 (951/878)	USA	Single center routine examination, BMI P5-P95, normal cardiac anatomy	Cycle	Ramp (10–25 W/min)	Exhaustion	Trajectories and formula (fractional polynomial regression)	Age, sex, BMI, race	Not readily usable in practice
Szmigielska et al., 2016 (32)	10–18 y	711 (457/254)	Poland	Athletes, single center, resting BP < P90	Cycle	Individual (multi stage 30/60 W every 3 min)	Exhaustion	Diagram (multivariate linear regression) depicting 2 SEE	Age, sex, workload	No adjustment for height
Hacke and Weisser, 2016 (33)	12–17 y	492 (251/241)	Germany	6 public schools no diagnosis of hypertension or CV disease	Cycle	Individual (multi-stage 0.5 W/kg every 3 min)	Submaximal 1.5 W/kg	P95 and P90 of SBP	Age, sex	No adjustment for height
Wanne and Haapoja, 1988 (60)	9–18 y	497 (260/237)	Finland	Random sample from multi-center study (*n* = 14.487) corrected for age and social class	Cycle	Ramp (HR controlled, increment of 8 bpm)	HR of 170 bpm	Trajectories with 2SD adjusted	Sex, HR, puberty (9–12 y/14–18 y)	Age group 12–14 y not reported

BMI, body mass index; bpm, beats per minute; CV, cardiovascular; DBP, diastolic blood pressure; f, female; HR, heart rate; m, male; N, number; P, percentile; SBP, systolic blood pressure; SD, standard deviation; SEE, standard error of estimate; W, watt; y, years.

Cycle ergometers are most frequently used in pediatric practice since they are cheaper, take up less space, and are easier to use, especially in the case of individuals with weight-bearing limitations (31). The downside of using a cycle ergometer is that muscle fatigue of the lower extremities can lead to the test ending prior to obtaining values, which are caused by central factors. In addition, for some children maintaining a constant rhythm of pedaling (cadence) can be a problem. Two relatively recent studies reported the BP readings of adolescents during laboratory testing conditions that involved a cycle ergometer (32, 33). Hacke and Weisser used a standardized graded submaximal exercise test at a Physical Working Capacity 170 (PWC 170) with a load of 1.5 W/kg body mass, while for participants who were overweight/obese corrections were made based on the average weight values for sex and age (33). The authors provided systolic resting BP and BP after exercise in sex- and age-related percentiles for the ages of 12–17 years. Szmigielska et al. presented data on BP during submaximal cycle ergometer testing among individuals aged 10–18 years, who were involved in some form of sports activity. Unfortunately, without any data on duration and intensity of the sports training and without determination of maximum oxygen uptake (32).

Laboratory treadmill testing is the method of choice for all functional testing as it relies on the well-known mechanics of walking and enables longer activity due to the smaller impact of local fatigue factors. However, data on BP for children and adolescents using this kind of testing are even more infrequent. Still, two new studies offer some normative values (31, 34). Sasaki et al. (34) measured BP and maximum oxygen uptake during a standardized test following the Bruce protocol (35). Systolic BP under load was presented in sex- and age-related percentiles for the ages of 7–17 years. A limitation of their study is that height of the participants and the values of resting BP were not taken into consideration. Clarke et al. (31) took matters a step further as they reported normative values for systolic BP during standardized testing under the Bruce protocol among children and adolescents aged 6–18 years by taking into consideration the participants’ height and resting BP (31). Their study explicitly excluded obese individuals because of anticipated differences in BP changes during physical activity in this specific group.

All of the studies on reference values have their limits as summarized in Table 1. In the absence of more complete reference values, the percentiles proposed by Sasaki et al. (34) (treadmill) and Hacke and Weisser (33) (cycle) may be considered the most practical. An overview of the respective 95th percentile as a cut off for clinical use is provided in Supplementary Table 1.

## Exaggerated blood pressure response

Some individuals present with abnormally exaggerated rise in systolic BP during exercise. This phenomenon is known as an exaggerated blood pressure response to exercise (EBPR). It can be observed in individuals without known CV diseases. EBPR can be explained by impairment of endothelial function with an impairment of exercise induced endothelial vasodilation, especially in younger individuals. Other possible mechanisms of EBPR could be an augmented rise of angiotensin II during exercise found in individuals with EBPR (36). EBPR is generally considered to be a pathological response, however, its clinical significance is not entirely clear.

Exaggerated blood pressure response is defined as an increase in BP (with or without a correlation to heart rate) that is too high for the individual undergoing (sub)maximal stress test. In adults, classically cut off points of >210 mm Hg in men and >190 mm Hg in women are proposed based on the CARDIA study (37). However, even in adults, the definition of EBPR is hampered by differences in methodology and criteria used during testing, e.g., whether the exercise is performed on a treadmill or bicycle, and the BP is measured at a moderate or a maximum level of exercise (38).

As indicated in the previous section, the definition of exaggerated blood pressure in the pediatric population is related to sex- and age-specific reference values and not to fixed cut-off points. It does not come as a surprise that the differences in methodology and criteria as described for adults result in different reference values for the pediatric population, which makes the interpretation of stress testing difficult. Only a limited number of studies examined the levels of post-exercise SBP in the general population. Data from the above-mentioned German population-based study on 531 healthy adolescents aged 12–17 years showed that 13.6% of the adolescents had at least high normal exercise SBP values and 5.9% displayed both, increases in resting as well as end exercise BP. Moreover, in this study 7.7% of children had increased resting but normal exercise BP (33). The retrospective study from Michigan, USA, based on 1,085 children aged 7–17 years showed that up to 14.1% of children presented with an exaggerated BP after treadmill exercise (34). As indicated, the strength and limitation of those studies is using centiles—relative measure in contrary to fixed mmHg values. This will finally need to be reconsidered as using this cut-off point will always give the risk of about 5% of inappropriate results. Contrary, using fixed cut-off points (ex. adult) can give both, over- or underestimating the true number of affected children.

## Exaggerated blood pressure response indicating future hypertension development

High BP is a leading risk factor for CV disease (CVD). The identification of high BP is conventionally based on in-clinic (resting) BP measures, performed within primary health care settings. However, many cases of high BP go unrecognized or remain inadequately controlled. Thus, there is a need for complementary settings and methods for BP assessment to identify and control high BP more effectively. Since the early 1980s, numerous studies made on adults, have shown a relationship between the presence of exaggerated BP response to exercise and the future risk of developing HTN. However, many of these studies were conducted on small samples and were not representative of the general population (39). The two main population-based studies in which it was identified that exaggerated blood pressure response to exercise could be a predictor of the development of hypertension were the CARDIA study (687 subjects followed for 5 years) and a Framingham study sample of 2,310 participants who were followed for 8 years (37, 40).

Keller et al. recently published a systematic review of 18 original studies of retrospective and prospective design including 35,151 healthy normotensive adult subjects (41). The follow-up period varied between 2 and 14 years. Most studies showed an association between EBPR (systolic, diastolic, or both) and incident HTN regardless of the heterogeneity of the criteria used to define the hypertensive response. Therefore, in adults, it could be concluded that EBPR is able to identify a subgroup of patients at high risk for developing resting HTN.

Forty years ago, some small studies made on adolescents found that SBP during exercise was significantly higher in subjects with a parental history of HTN compared with those without (42, 43). These results suggest that the exaggerated BP responses to exercise, characteristic of hypertensive patients, may be present in normotensive adolescents with an increased risk of developing the disorder, and may reflect pathophysiological changes that precede sustained BP elevation. A sub-study of the European Youth Heart Study aimed to analyze whether SBP, heart rate, and rate pressure product (RPP) measured during exercise in childhood could predict resting SBP levels in adolescence independent of resting SBP and conventional CV risk factors (44). This was studied in a sample of 226 randomly selected children followed longitudinally for 6 years and re-assessed during adolescence. SBP and rate pressure product during exercise in stage two of the test were positively associated with future resting SBP, independent of resting SBP in childhood. After additional adjustment for conventional CV risk factors, the associations with SBP and rate pressure product during stage two on future resting SBP changed only slightly with rate pressure product remaining significant (*P* = 0.059 and *P* = 0.012, respectively). Rate pressure product expressed as the product of SBP and heart rate during exercise was associated with future BP levels. Based on these results, it could be inferred that measuring BP during exercise might be of diagnostic value in some children at risk for developing hypertension. This is not only the case in children with a family history of HTN, but also for certain clinical conditions such as obesity, heart and kidney disease, for which an association to exercise-induced increases in BP have been proposed (45–48). As EBPR increases the risk of developing future HTN, these children would benefit from an early detection of EBPR resulting in a closer BP control. This would be of specific importance during adolescence and just before transitioning to adulthood, a time of life when systematic health check-ups end. Nevertheless, there are no prospective data available on the real impact of EBPR in childhood and the risk of developing HTN in adulthood (33). The European Society of Hypertension guidelines for the management of high BP in children and adolescents, therefore, do not recommend exercise testing in this specific setting (49). On the other hand, the same guidelines recommend ambulatory BP measurement (ABPM) in those subjects with an EBPR.

This later recommendation is based on studies such as the one by Kavey et al. (50). They evaluated 119 children aged 6–18 years with confirmed office HTN. Office BP, ABPM, and BP response to treadmill exercise were measured. They observed an exaggerated SBP response to exercise not only in 61% of the boys and 64% of the girls in the HTN group (HTN based on office BP and ABPM), but also in 39% of boys and 36% of girls from the group of white-coat hypertensive subjects (office HTN, but normal ABPM) (50). Moreover, an exaggerated SBP exercise response predicted HTN on ABPM in 63%, while a normal SBP response to exercise had a negative predictive value of 72%. This association between EBPR and elevated ABPM was confirmed in the Avon Longitudinal Study of Parents and Children (ALSPAC) (51). In the ALSPAC, a total of 657 adolescents (mean age: 17.7 ± 0.3 years; 42% male) completed a step-exercise test with pre-, post-, and recovery-exercise BP, office BP and ABPM. Fifty participants (7.8%) were classified with masked hypertension. Office BP, pre-, post-, and recovery-exercise systolic BP were associated with masked hypertension (AUC ≥ 0.69 for all, respectively), with the office systolic BP threshold of 115 mm Hg having high sensitivity and specificity and exercise BP thresholds of 126, 150, and 130 mm Hg, respectively, having high specificity and negative predictive value (individually or when combined) for ruling out the presence of masked hypertension. Based on these results, the authors concluded that “systolic BP responses to step-exercise testing, not being definitive in terms of “diagnosing” masked hypertension (which should be confirmed with out-of-clinic BP monitoring), the high negative predictive values indicate that the presence of masked hypertension could be effectively ruled out by exercise testing. This observation is important as individuals found to have normal office BP have no indication for an out-of-clinic BP monitoring. Thereby, the presence of masked hypertension may be overlooked and the associated CV risk missed. The measurement of BP during exercise could be therefore a good screening tool.

## Exaggerated blood pressure response and its association with cardiovascular risk factors

Other studies have found children with high LDL-cholesterol levels to have significantly higher SBP and DBP immediately before and after a treadmill exercise, as well as at the end of post-exercise recovery (52). These findings were observed in children with severely elevated levels of LDL-cholesterol suggesting that severely unfavorable levels of blood lipids are necessary to impair the regulation of BP during exercise. The association between conventional CVD risk factors and BP response during acute exercise was studied in 439 Danish third-grade children and 364 ninth-grade adolescents who participated in the European Youth Heart Study (53). Researchers found that HOMA-IR score was positively associated with SBP response during exercise in boys. This relationship remained significant after controlling for resting SBP, exercise heart rate, body height, school grade, test protocol, and workload. In boys, they also found a significant positive relationship between BMI and SBP response.

While various studies suggested an EBPR in obese children (47), a small but very elegant study Dipla et al. (54) showed that after handgrip exercise obese children without a family history of hypertension did not show an exaggerated blood pressure response. Notably, the obese and non-obese children in this study had equal blood pressure levels at baseline. In this study, obese and lean children showed different adaptions to exercise: the obese children increased stroke volume whereas the lean children increased systemic vascular resistance.

## Exaggerated blood pressure response and its association with target organ damage and cardiovascular disease

An exaggerated SBP during exercise has also been reported in children at risk for early damage in vascular structure and functioning. This was observed in the study by Kavey et al. (50), in which LV mass correlated significantly with office SBP, maximal exercise SBP (SBP max), ABPM awake and asleep SBP. Using multiple regression, maximum correlation was achieved with inclusion of height, weight, ABPM awake SBP, and treadmill exercise (TE) maximal SBP. Previously similar results have been observed in a bigger cohort of 274 subjects (6–15 years) based on the Muscatine study (55). Anthropometrical variables, office BP, BP response to exercise in an ergometer, and echocardiographic left ventricular mass (LVM) were assessed at baseline and after a mean of 3.4 years. At baseline, LVM correlated with maximum SBP during exercise and also with SBP increase during exercise. The final SBP was best predicted from the initial variables of office SBP, SBP max, and LVM; and the final LVM was best predicted from the initial variables of LVM and DBP max. Assessment of left-ventricular mass index (LVMI) and carotid-femoral pulse wave velocity (aortic PWV) was also undertaken in the ALSPAC cohort (51). The proposed cut-off points for pre-, post-, and recovery-exercise systolic BP were associated with greater LVMI and aortic PWV. These results indicate that detecting EBPR could identify children and adolescents at risk for the development of target organ damage (TOD).

There is evidence that EBPR predicts CV events and CV mortality in otherwise normotensive adults (56, 57). On the other hand, post-exercise hypotension, defined as a drop of SBP below pre-exercise values, is often indicative of significantly increased risk for cardiac events, especially when associated with prior myocardial infarction or exercise-induced ischemia (58). There is no information on the prognostic value of these measurements in children and adolescents, but a hypotensive response to exercise is a criterion to end exercise testing in children (48).

## Conclusion

**FIGURE 2:**
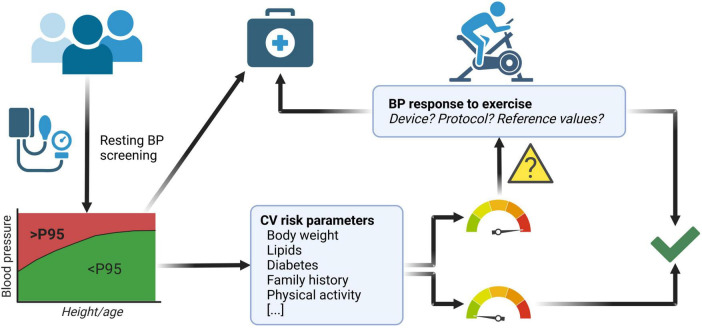
Possible implementation of exercise stress testing to improve cardiovascular (CV) health screening in children and adolescents. Measurement of resting blood pressure (BP) identifies children and adolescents with elevated BP [i.e., pathologic reading above the 95th percentile according to guidelines (49)], who can be subsequently referred to medical follow up and potential treatment. However, subjects with a normal resting BP (i.e., below the 95th percentile) but presenting with additional CV risk factors (e.g., obesity/adiposity, abnormal lipid status, abnormal glucose tolerance or overt diabetes, a family history of CV disease and/or a severe lack of physical activity) may profit from further evaluation by undergoing exercise stress testing. An exaggerated BP response during exercise stress testing would warrant closer medical follow up. Children and adolescents with normal BP response to exercise as well as those with no other CV risk factors do not need increased medical attention. When performing exercise stress testing one must be aware of the limitations described in this article with regard to device, protocol and reference values. Figure created with BioRender.com.

In this review, we discuss the potential usefulness of stress testing to unmask EBPR, especially in children presenting with certain risk factors this diagnostic procedure might be useful (Figure 2). In adulthood the link between EBPR and CV events and mortality has been demonstrated. The situation during childhood is less clear due to a lack of studies showing that EBPR is preceding HTN and CV morbidity. In addition, studies performing stress testing are hampered by methodical limitations inherent to the currently available reference values. Still, it might be reasonable to have children undergo exercise stress testing as a rather non-invasive procedure to add additional information with regard to their CV risk profile. But at the moment there is not enough data to make the general recommendation of introducing BP measurement during exercise in the cardiovascular risk disease evaluation in children and adolescents. Future studies are needed that use similar methodology to extend our current knowledge on possible links between the presence of certain clinical conditions, the detectability of an EBPR and the risk of developing CV morbidity and mortality in later life.

## Author contributions

All authors listed have made a substantial, direct, and intellectual contribution to the work, and approved it for publication.
