# Supplementary material for: Blood pressure response to exercise in children and adolescents
Source: Front Cardiovasc Med. 2022 Sep 30;9:1004508. doi: 10.3389/fcvm.2022.1004508 (PMC9561233; doi:10.3389/fcvm.2022.1004508)
Supplement: Supplementary file 1 [file Table_1.docx]

BP response to exercise in children and adolescents

Julio Alvarez-Pitti^1,2,3^, Vesna Herceg-Čavrak^4^, Małgorzata Wójcik^5^, Dragan Radovanovic^6^, Michał Brzeziński^7^, Carl Grabitz^8^, Elke Wühl^9^, Dorota Drożdż^10^*^†^, Anette Melk^8^*^†^

^1^ Pediatric Department, Consorcio Hospital General, University of Valencia, Valencia, Spain

^2^ CIBER Fisiopatología Obesidad y Nutrición (CIBEROBN), Instituto de Salud Carlos III, Madrid, Spain

^3^ INCLIVA Biomedical Research Institute, Hospital Clínico, University of Valencia, Valencia, Spain

^4^ Libertas International University, Croatia

^5^ Department of Pediatric and Adolescent Endocrinology, Chair of Pediatrics, Pediatric Institute, Jagiellonian University Medical College, Cracow, Poland

^6^ Department of Medical Sciences, Faculty of Sport and Physical Education, University of Nis, Nis, Serbia

^7^ Department of Pediatrics, Gastroenterology, Allergology and Pediatric Nutrition, Medical University of Gdansk, Gdansk, Poland

^8^ Children’s Hospital, Hannover Medical School, Hannover, Germany

^9^ Division of Pediatric Nephrology, Center for Pediatrics and Adolescent Medicine, Heidelberg University Hospital, Heidelberg, Germany

^10^ Department of Pediatric Nephrology and Hypertension, Pediatric Institute, Jagiellonian University Medical College, Cracow, Poland

^†^ These authors contributed equally to this work and share senior authorship

**Correspondence:**

Anette Melk, MD PhD; Children’s Hospital, Hannover Medical School, Carl Neuberg Str. 1, 30625 Hannover, Germany; Email: [melk.anette@mh-hannover.de](mailto:melk.anette@mh-hannover.de); Phone: +49-511-532 5597

Dorota Drożdż, MD; Jagiellonian University Medical College, Department of Pediatric Nephrology and Hypertension, 265 Wielicka Str, 30-663 Krakow, Poland; Email: [dorota.drozdz@uj.edu.pl](mailto:dorota.drozdz@uj.edu.pl); Phone: +48 604787415

**Keywords**: Blood pressure, exercise, stress test, children, adolescents

**Supplement:**

**Table S1:** P95 for systolic blood pressure (in mmHg) by age group during pediatric exercise testing

|  | Treadmill  Sasaki et al. 2021 (1)  modified Bruce protocol | | Cycle  Hacke et al. 2016 (2)  multi stage, submaximal 1.5W/kg | |
| --- | --- | --- | --- | --- |
| Age (years) | Girls | Boys | Girls | Boys |
| 7 | 174 | 167 |  |  |
| 8 | 175 | 171 |  |  |
| 9 | 177 | 176 |  |  |
| 10 | 178 | 180 |  |  |
| 11 | 179 | 185 |  |  |
| 12 | 181 | 189 | 172 | 172 |
| 13 | 182 | 194 |  |  |
| 14 | 184 | 198 | 174.4 | 177.3 |
| 15 | 185 | 203 |  |  |
| 16 | 186 | 207 | 178.5 | 201.3 |
| 17 | 188 | 212 |  |  |

# References

1. Sasaki T, Kawasaki Y, Takajo D, Sriram C, Ross RD, Kobayashi D. Blood Pressure Response to Treadmill Cardiopulmonary Exercise Test in Children with Normal Cardiac Anatomy and Function. *J Pediatr* (2021) 233:169-74.e1. Epub 2021/02/26. doi: 10.1016/j.jpeds.2021.02.043.

2. Hacke C, Weisser B. Reference Values for Exercise Systolic Blood Pressure in 12- to 17-Year-Old Adolescents. *Am J Hypertens* (2016) 29(6):747-53. Epub 2015/11/14. doi: 10.1093/ajh/hpv178.
